# Characterization of Indigenous Bacteria for Microbially Induced Carbonate Precipitation in a Limestone Mine

**DOI:** 10.3390/microorganisms13091985

**Published:** 2025-08-26

**Authors:** Xiulun Shen, Kimihiro Hashiba, Tomoyoshi Yakata, Kotaro Yoshida, Hajime Kobayashi

**Affiliations:** 1 Department of Systems Innovation, School of Engineering, The University of Tokyo, Tokyo 113-8656, Japan; shen-xiulun888@g.ecc.u-tokyo.ac.jp (X.S.); hashiba@sys.t.u-tokyo.ac.jp (K.H.); 2 Shinko Holdings Corporation, Tokyo 106-0041, Japan; t.yakata@shinko-hd.co.jp (T.Y.);; 3 Engineering for Sustainable Development of Subsurface Environments (Shinko Holdings Corporation) Social Cooperation Program, Department of Systems Innovation, School of Engineering, The University of Tokyo, Tokyo 113-8656, Japan

**Keywords:** biocementation, urea hydrolysis, low-temperature environments, calcite, *Rhodococcus*

## Abstract

Microbially induced carbonate precipitation (MICP) refers to the formation of calcium carbonate driven by microbial metabolic processes, such as ureolysis. As an emerging biocementation technique, MICP has garnered attention for various applications in environmental and civil engineering. This study evaluated the feasibility of MICP implementation in a limestone mine. Ureolytic bacteria were isolated from an active limestone quarry at Mt. Buko, Saitama, Japan. Located at an elevation above 1000 m, the site represents a low-temperature environment with an average annual temperature of ~10 °C. The representative isolates, *Rhodococcus* sp. strains L6 and L8, exhibited tolerance to key environmental factors relevant to MICP applications in the limestone-rich settings, including alkaline pH, high calcium levels, and elevated urea concentrations. Additionally, both strains were psychrotolerant, maintaining growth and urease activity at temperatures as low as 5 °C. Notably, both strains induced calcite crystal formation at 10 °C and 5 °C, although the reaction was slower at 5 °C. Furthermore, strain L6 demonstrated the ability to induce MICP on limestone surfaces, effectively sealing rock fissures. These findings suggest that indigenous microbes retain metabolic activity in the limestone mine and are well suited for MICP applications.

## 1. Introduction

Microbially induced carbonate precipitation (MICP) refers to the formation of CaCO_3_ through various microbial metabolic activities, including urea hydrolysis [1,2,3,4], denitrification [5,6,7,8], sulfate reduction [9], iron reduction [10,11], methane oxidation [12], and photosynthesis [5,13]. Among these, MICP via urea hydrolysis has been most extensively studied. In this pathway, ureolytic bacteria hydrolyze urea into ammonium and CO_2_, considerably increasing local pH and carbonate ion concentration. The bacterial cell surface and extracellular polymeric substances (e.g., extracellular polysaccharides) serve as nucleation sites for mineral crystallization, promoting CaCO_3_ crystal precipitation in the presence of sufficient calcium ions [2,14,15].

MICP forms durable CaCO_3_ deposits from aqueous fluids with relatively low environmental impact. In soils, CaCO_3_ precipitates bind to and between soil particles, forming solid interparticle connections. Similarly, CaCO_3_ blocks pore throats and seals small fractures in porous materials, including rock and concrete. As a result, MICP effectively reduces media permeability while increasing mechanical strength. Additionally, CaCO_3_ can co-precipitate hazardous contaminants (e.g., toxic metals), limiting their mobility and bioavailability. Consequently, MICP has a wide range of applications in construction and environmental engineering, including soil and material improvement [4,11,12,16,17,18], restoration of natural sites and cultural artifacts, and water purification [10,14,19,20,21,22].

MICP is also applicable in mineral resource extraction. It can stabilize and reduce permeability in soil and rock within mining environments [23,24]. The low viscosity of MICP-promoting fluids allows deeper penetration into porous media. Therefore, MICP can reinforce subsurface soil or rock layers that cementitious grouts cannot reach. As a complementary method to traditional grouting, MICP may help prevent slope and quay wall failures, repair mining-induced fissures, control dust, and mitigate surface erosion. It has also been proposed for bioremediation of solid mining waste (e.g., ore and waste rock stockpiles, tailings, and slag) [25]. Such waste often contains harmful contaminants (e.g., heavy metals, arsenic, cyanide, and organics) and is typically stored in nearby environments, such as ponds or dams, where it is prone to oxidation and leaching into surrounding soil and groundwater. MICP can enhance the structural integrity of waste piles by consolidating solids, reducing the risk of landslides and erosion. It can also limit contaminant migration by lowering permeability and immobilizing contaminants through mineral precipitation. Additionally, suppression of coal mine dust via ureolysis-driven MICP has been investigated [26,27].

Numerous laboratory studies have shown that MICP can enhance mechanical strength and reduce permeability in various porous media, including soils and fractured or porous rocks [23,24]. For example, Gao et al. [28] demonstrated that ureolysis-driven MICP effectively sealed fractures in 3D-printed models of coal mine fractures. Furthermore, MICP has been explored for mine waste remediation by modifying its biochemical and geophysical properties (reviewed by Wilcox et al. [25]). Specifically, ureolysis-driven MICP can immobilize hazardous metals (e.g., arsenic, cadmium, copper, lead, strontium, and zinc) present in mine waste [29,30,31,32,33,34,35].

Most prior studies have assessed MICP under standard laboratory conditions. However, mining environments are highly variable and often harsh for microbial activity (e.g., extreme pH, wide ion concentration ranges, and temperature extremes), posing challenges for MICP implementation. In this context, some studies have indicated that indigenous microorganisms from mine sites may be better suited for in situ applications, as they are naturally adapted and tolerant to their local conditions [28,30,32,33,34,35].

Accordingly, we conducted this proof-of-concept study to evaluate the feasibility of using indigenous microorganisms for ureolysis-driven MICP in mining environments. We isolated ureolytic bacteria from an active limestone mine and examined their tolerances and MICP activity under site-relevant conditions, particularly low temperatures (10 °C and 5 °C), reflecting the mine’s environmental conditions.

## 2. Materials and Methods

### 2.1. Sample Collection

In October 2024, environmental samples were collected from a limestone mining area in Mt. Buko (Saitama, Japan). Bare soil was sampled in a quarry located at an altitude of ~1000 m. Limestone mine dust sedimented on mineshaft surfaces near a rock crusher was also collected. Samples were collected using sterilized spoons, placed into sterile 50 mL tubes, and stored at 4 °C until inoculation.

### 2.2. Screening and Isolation of Ureolytic Bacteria

Christensen urea medium is a differential medium containing the pH indicator phenol red. Urease-producing bacteria hydrolyze urea, releasing ammonia and raising pH, which turns the medium’s color from orange to pink. Samples were suspended in sterilized saline [0.85% (*w*/*v*) NaCl] and serially diluted 10-fold (1–10^4^ dilutions). Subsequently, 100 µL of each dilution was spread onto Christensen urea agar plates (1.0 g/L peptone, 1.0 g/L glucose, 20.0 g/L urea, 5.0 g/L NaCl, 2 g/L KH_2_PO_4_, 0.012 g/L phenol red, and 15.0 g/L agar [36]); urea and glucose were filter-sterilized (0.2 µm pore size) and added separately after autoclaving. Plates were incubated aerobically overnight at ambient temperature (~18 °C). Urease-positive colonies were streaked onto fresh plates to isolate single colonies, with this process repeated three times to ensure purity.

### 2.3. Phylogenetic Characterization of the Isolates

Following overnight culturing in nutrient broth (1 g/L beef extract, 5 g/L peptone, 5 g/L NaCl, and 2 g/L yeast extract), genomic DNA was extracted using a DNeasy PowerSoil Kit (Qiagen, Hilden, Germany). DNA was amplified via PCR using Ex Taq DNA polymerase, hot-start version (Takara Bio Inc., Kyoto, Japan), following the manufacturer’s instructions as well as the 8F (5′-AGAGTTTGATYMTGGCTCAG-3′) and 1492R (5′-CGGYTACCTTGTTACGACTT-3′) primers to target nearly the full length of the 16S rRNA gene [37]. Thermocycling involved an initial denaturation at 95 °C for 5 min, 40 cycles of 1 min at 95 °C, 1 min at 50 °C, and 2 min at 72 °C, followed by a final extension at 72 °C for 10 min. Amplicons were sequenced by Macrogen Japan (Tokyo, Japan) using Sanger sequencing. In December 2024, the 16S rRNA gene sequences were compared against sequences in the NCBI nonredundant nucleotide database (nr/nt) using blastn (https://blast.ncbi.nlm.nih.gov/Blast.cgi, accessed on 16 December 2024). A phylogenetic tree was constructed using MEGA version 12 [38], applying the Tamura–Nei model and neighbor-joining method, with 500 bootstrap replicates employed to assess node confidence.

### 2.4. Carbonate Precipitation Test

Isolates were streaked onto Luria–Bertani agar plates (10 g/L tryptone, 10 g/L NaCl, 5 g/L yeast extract, and 15 g/L agar) from glycerol stocks stored at −80 °C and incubated overnight at 30 °C. A fresh colony was inoculated into 10 mL of nutrient broth (1 g/L beef extract, 5 g/L peptone, 5 g/L NaCl, and 2 g/L yeast extract) supplemented with 17.6 g/L CaCl_2_ and incubated overnight at 30 °C with shaking (190 rpm). This preculture was then inoculated into a baffled 250 mL Erlenmeyer flask containing 30 mL of the same broth supplemented with 17.6 g/L CaCl_2_ and 20 g/L urea, to an initial optical density at 600 nm cm^−1^ (OD_600_) of approximately 0.001. Cultures were incubated with shaking (190 rpm) at 5 °C, 10 °C, or 30 °C for 7 d (10 °C and 30 °C) or 14 d (5 °C). OD_600_ was measured using an Ultrospec 6300 Pro spectrophotometer (GE Healthcare, Chicago, IL, USA). Culture pH and electrical conductivity (EC) were measured using a SevenExcellene S470-Basic pH/EC meter (Mettler Toledo, Greifensee, Switzerland). Insoluble precipitates were collected from the cultures via centrifugation, washed three times with distilled water and 100% (*v*/*v*) ethanol, and dried at 70 °C for 24 h. The dry weight of precipitates was measured to estimate the precipitate formation. Experiments were performed in triplicate and included uninoculated controls.

### 2.5. Assessment of Effects of Various Environmental Conditions on Growth and Urease Activity of Strains L6 and L8

Strains L6 and L8 were streaked onto Luria–Bertani agar plates from glycerol stocks stored at −80 °C and incubated overnight at 30 °C. A fresh colony was inoculated into 10 mL of nutrient broth and incubated overnight at 30 °C with shaking (190 rpm). This preculture was then inoculated into a baffled 250 mL Erlenmeyer flask containing 30 mL of the nutrient broth or modified nutrient broths: To assess the effect of culturing temperatures, the nutrient broth was used (5 °C–40 °C in 5 °C intervals); to assess the effect of initial pH, the initial pH of the nutrient broth was adjusted to 4.0, 5.0, 6.0, 7.0, 8.0, 9.0, 10.0, 11.0, 11.5, 12.0, 12.5, and 13.0 by adding HCl or NaOH; to assess the effect of salinities, the NaCl concentrations of nutrient broth were adjusted to 0%, 5%, 10%, 15%, and 20% (*w*/*v*); to assess the effect of urea concentrations, 0.25, 0.5, 0.75 and 1.0 mol/L urea were added to the nutrient broth; to assess the effect of CaCl_2_, 0.25, 0.5, 0.75, 1.0, 1.25, and 1.5 mol/L CaCl_2_ were added to the nutrient broth. The cultures were incubated for 48 h at 30 °C (except for the tests at different temperatures) with shaking (190 rpm). Urease activity of the culture was determined using the conductivity method [39]: 1 mL of the culture was mixed with 9 mL of 1.0 mol/L urea solution. The change in EC of the mixture was measured as described above at 25 °C within 5 min. The average rate of EC change ((mS/cm)/min) was then converted to the urease activity (urea hydrolysis rate) (mM/min) by multiplying by the urease activity coefficient (11.11 at 25 °C). OD_600_ was measured as described above. Culture pH was measured as described above at 48 h post-inoculation. Experiments were performed in triplicate.

### 2.6. Mineralogical and Morphological Analyses of the Precipitates

Mineralogical properties of the dried precipitates were analyzed using Fourier transform infrared (FTIR) spectroscopy, X-ray diffraction (XRD), field emission scanning electron microscopy (SEM), and energy-dispersive X-ray spectroscopy (EDS). FTIR was performed using an Alpha II compact FTIR spectrometer (Bruker Scientific, San Jose, CA, USA) across 400–4000 cm^−1^ with a resolution of 2 cm^−1^. XRD was performed using a RINT-2100 diffractometer (Rigaku Co., Tokyo, Japan) with copper Kα radiation (λ = 0.15406 nm), operated at 40 kV and 30 mA, scanning at 2°/min for 2θ values over a wide range of Bragg angles (20° ≤ 2θ ≤ 90°). SEM and EDS were performed using a JSM-7800F scanning electron microscope (JEOL, Tokyo, Japan) at a 10 kV accelerating voltage.

### 2.7. Rock Fissure Grouting Test

Cylindrical limestone cores from the Mt. Buko mine were used for the rock fissure grouting test. The circular faces (2.5 cm in diameter) were polished using a rock glider to ensure flat surfaces. Two cores were held face-to-face using rubber bands to simulate a rock fissure. Fresh preculture (1 mL) was pipetted between the cores (i.e., into the “rock fissure”). Each pair was submerged in nutrient broth containing 17.6 g/L CaCl_2_ and 20 g/L urea and incubated at 10 °C without shaking for 14 d. Air was continuously supplied to the medium using an air pump (200SB, Gex, Osaka, Japan). Control experiments without urea were also performed. After incubation, grouted cores were rinsed and dried at 70 °C for 3 d. Splitting tensile strength was measured using a digital force gauge (FGJN-5, Nidec Drive Technology, Kyoto, Japan). One core was clamped to the gauge while the other was fixed, and the cores were pulled apart to measure tensile strength. Each experiment was performed five times (*n* = 5).

## 3. Results

### 3.1. Isolation of Ureolytic Bacteria from Samples Collected at a Limestone Mine

**Figure 1:**
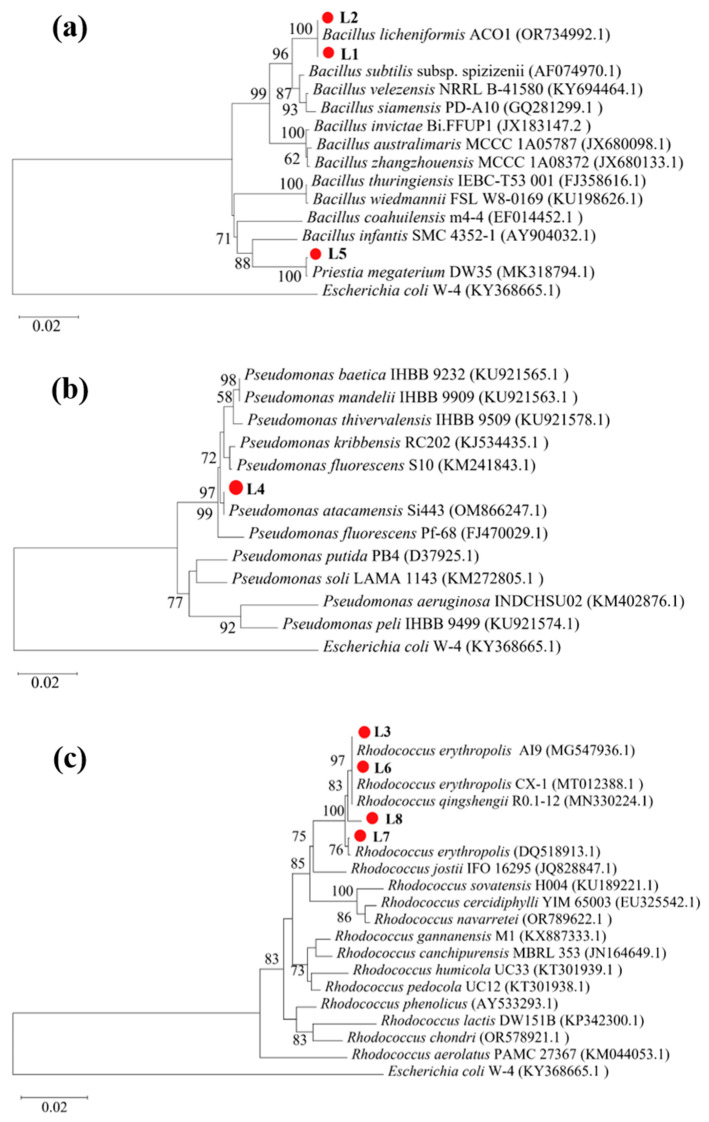
Phylogenetic relations of strains L1–L8 with reference strains. (**a**) L1, L2, and L5 with other *Bacillus* spp.; (**b**) L4 with *Pseudomonas* spp.; and (**c**) L3 and L6–L8 with *Rhodococcus* spp.

To identify bacteria capable of mediating ureolysis-driven MICP in limestone mining environments, we screened environmental samples (bare soil from the limestone quarry and limestone dust) collected from a limestone mine using Christensen urea agar plates. Six strains (L1–L6) were isolated from the quarry soil, with two (L7 and L8) isolated from the limestone dust; all eight exhibited ureolytic activity. Phylogenetic analysis of 16S rRNA gene sequences revealed that four strains (L3 and L6–L8) belonged to the genus *Rhodococcus*, three (L1, L2, and L5) to the genus *Bacillus*, and one (L4) to the genus *Pseudomonas* (Figure 1).

### 3.2. Assessment of the Isolates’ Ureolysis-Driven MICP Potential

**Figure 2:**
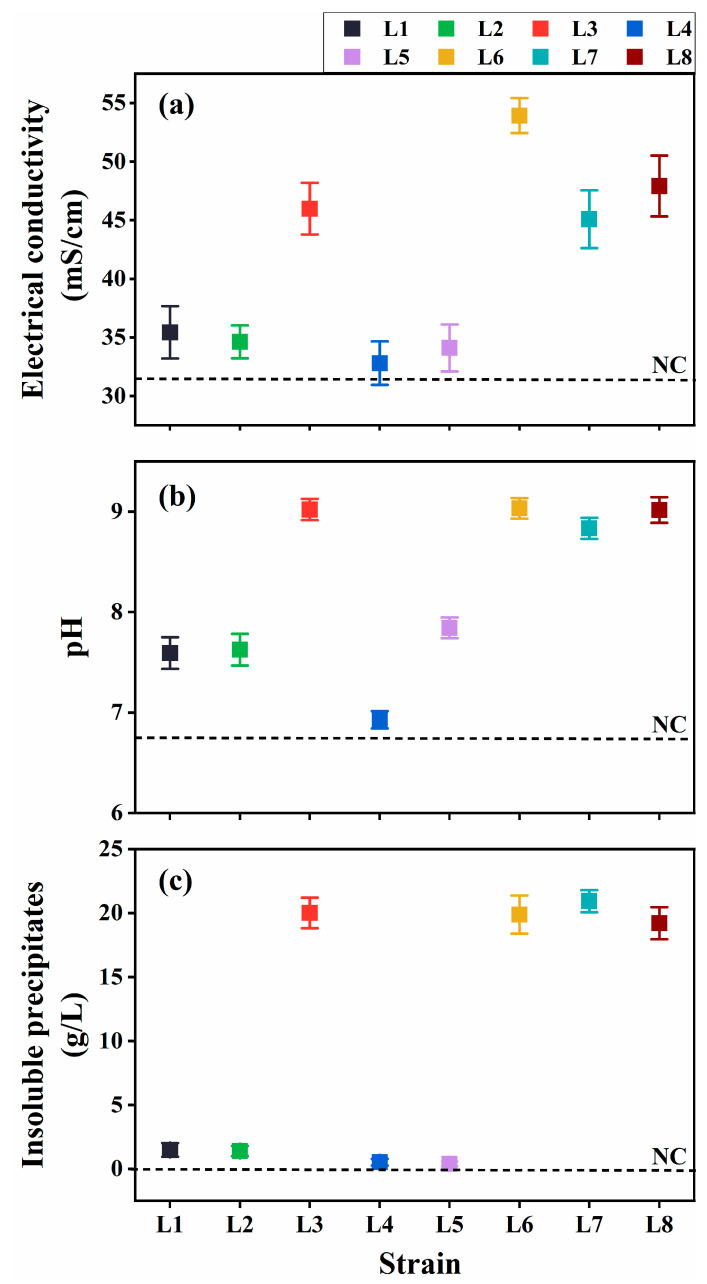
Ureolysis-driven microbially induced carbonate precipitation of strains L1–L8 at 30 °C. (**a**) Electrical conductivity (EC), (**b**) media pH, and (**c**) precipitate formation. NC, negative control.

The ureolytic activity and resulting MICP potential of the eight isolates were evaluated in the cultures at 30 °C at 7 d post-inoculation (dpi). The cultures inoculated with *Rhodococcus* strains (L3 and L6–L8) exhibited considerably higher EC and elevated pH compared with the uninoculated control (Figure 2a,b), indicating substantial ureolytic activity, which increased ion concentration and ammonia production. Aligning with these findings, notable insoluble precipitate formation was observed in the cultures at 7 dpi (Figure 2c).

**Figure 3:**
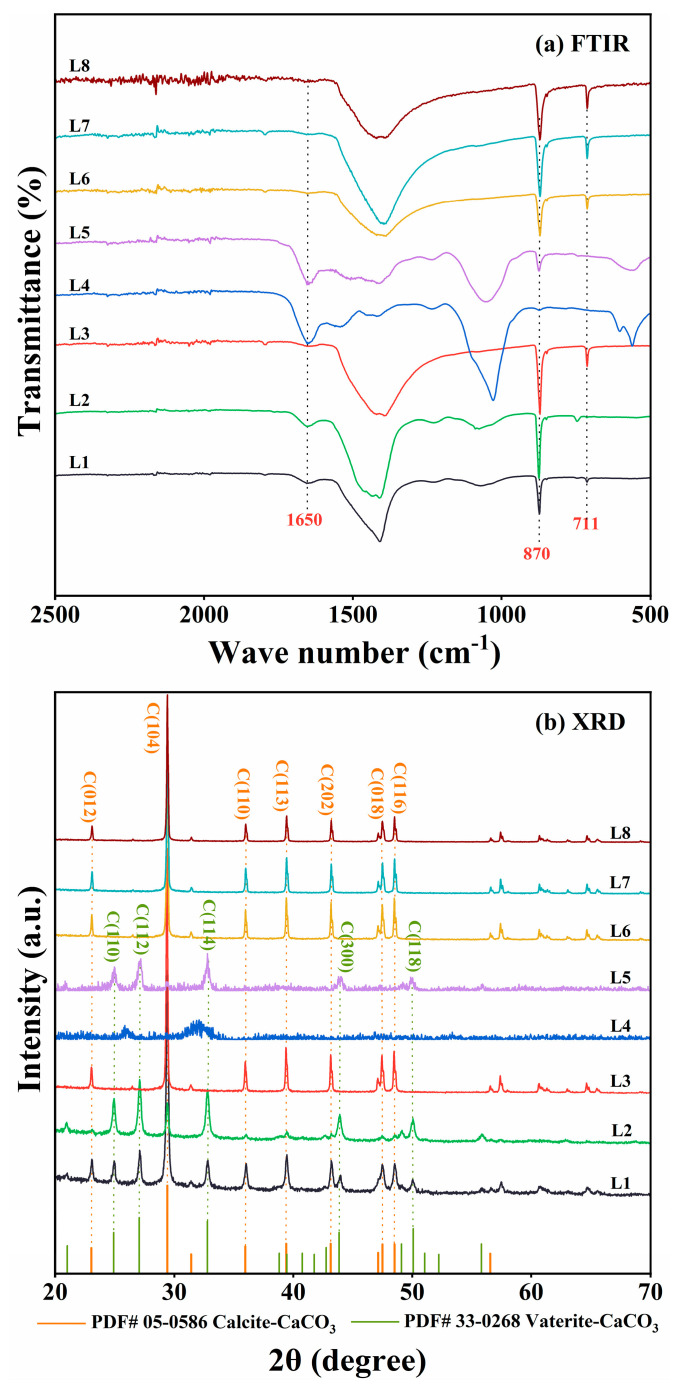
(**a**) Fourier transform infrared (FTIR) spectroscopy and (**b**) X-ray diffraction (XRD) analyses of mineralization products produced by strain L1–L8 cultures at 30 °C. In panel (**a**), only the FTIR spectra from 500 to 2500 cm^−1^ are shown, as the most notable differences were detected within the range. In panel (**b**), only the XRD spectra within the range of 20° ≤ 2θ ≤ 70° are shown, as the peaks corresponding to calcite and vaterite were detected within the range.

The precipitates were analyzed using FTIR and XRD. FTIR spectra of cultures with *Rhodococcus* strains (L3 and L6–L8) showed characteristic CaCO_3_ peaks at 870 and 711 cm^−1^ (Figure 3a). Consistent with these results, XRD analyses identified crystalline peaks characteristic of calcite in the same cultures (Figure 3b). In contrast, the other strains (L1, L2, L4, and L5) demonstrated weaker ureolytic activity and produced less precipitates. The FTIR spectra of these four strains displayed a peak at 1650 cm^−1^ corresponding to C=O stretching vibration, indicative of proteins or amides, and likely indicating the presence of biomass in the precipitates (Figure 3a). Moreover, XRD analyses indicated that strains L1, L2, and L5 produced both calcite and vaterite (Figure 3b). CaCO_3_ was not detected in the culture of strain L4. Based on these results, *Rhodococcus* strains L6 and L8, isolated, respectively, from quarry soil and limestone dust, were selected as representative ureolytic isolates for further investigation.

### 3.3. Assessment of Strain L6 and L8 Growth and Urease Activity Under Various Environmental Conditions

**Figure 4:**
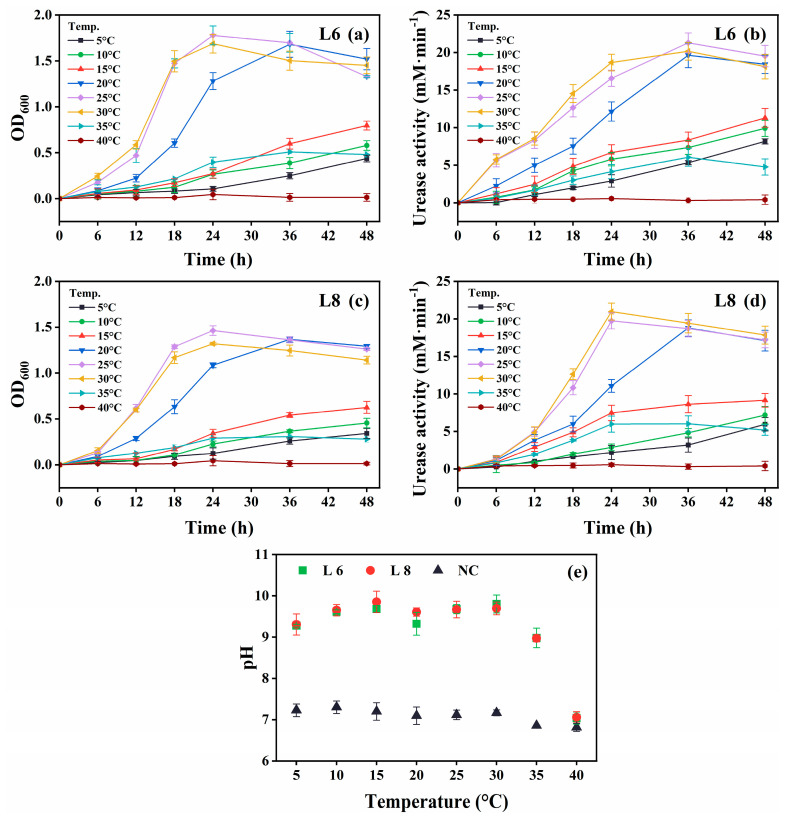
Effect of temperature on the growth and urease activity of strains L6 and L8 (initial pH of the medium, 7.0; NaCl concentration, 0.5%; no urea and CaCl_2_ were added to the cultures). (**a**) OD_600_ and (**b**) urease activity of strain L6. (**c**) OD_600_ and (**d**) urease activity of strain L8. (**e**) Media pH. NC, negative control; OD_600_, optical density at 600 nm.

**Figure 5:**
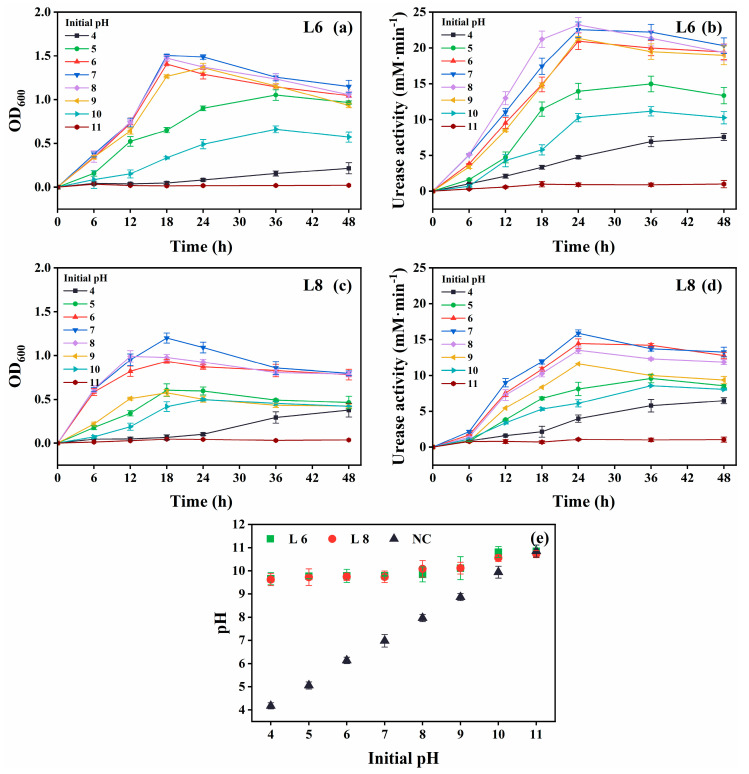
Effect of initial pH on the growth and urease activity of strains L6 and L8 (cultivation temperature, 30 °C; NaCl concentration, 0.5%; no urea and CaCl_2_ were added to the cultures). (**a**) OD_600_ and (**b**) urease activity of strain L6. (**c**) OD_600_ and (**d**) urease activity of strain L8. (**e**) Media pH. NC, negative control; OD_600_, optical density at 600 nm.

**Figure 6:**
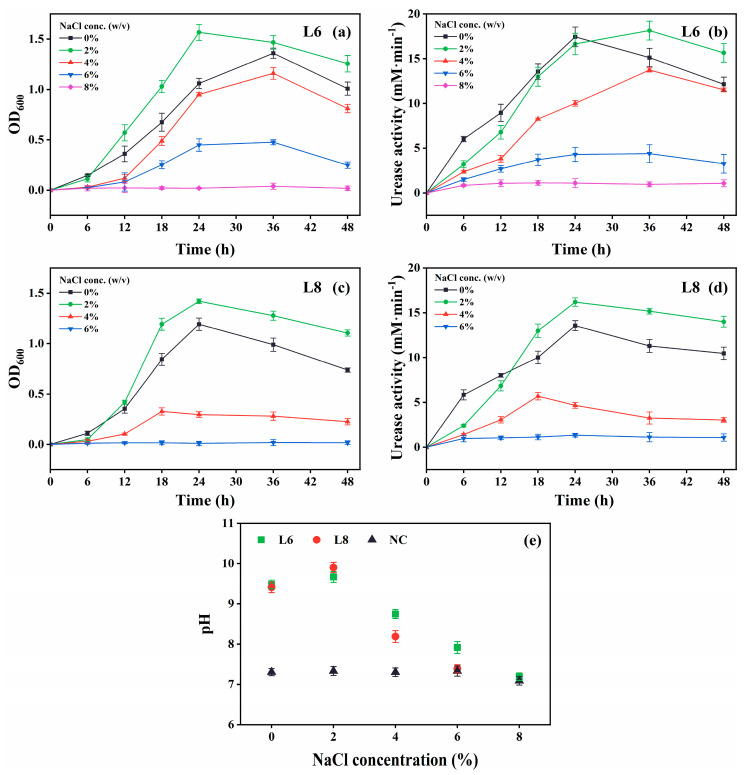
Effect of NaCl levels on the growth and urease activity of strains L6 and L8 (cultivation temperature, 30 °C; initial pH of the medium, 7.0; no urea and CaCl_2_ were added to the cultures). (**a**) OD_600_ and (**b**) urease activity of strain L6. (**c**) OD_600_ and (**d**) urease activity of strain L8. (**e**) Media pH. NC, negative control; OD_600_, optical density at 600 nm.

**Figure 7:**
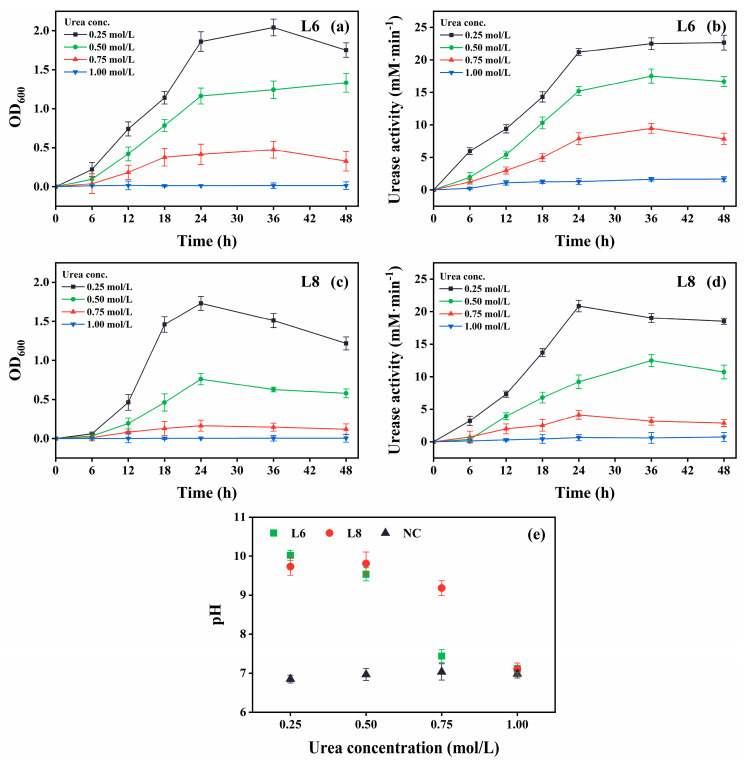
Effect of urea concentration on the growth and urease activity of strains L6 and L8 (cultivation temperature, 30 °C; initial pH of the medium, 7.0; no CaCl_2_ was added to the cultures). (**a**) OD_600_ and (**b**) urease activity of strain L6. (**c**) OD_600_ and (**d**) urease activity of strain L8. (**e**) Media pH. NC, negative control; OD_600_, optical density at 600 nm.

The growth and urease activity of strains L6 and L8 were evaluated under various environmental conditions: different temperatures (5 °C–40 °C in 5 °C intervals; Figure 4), initial pH values (4.0, 5.0, 6.0, 7.0, 8.0, 9.0, 10.0, 11.0, 11.5, 12.0, 12.5, and 13.0; Figure 5), salinities [0%, 5%, 10%, 15%, and 20% (*w*/*v*) NaCl; Figure 6], and urea concentrations (0–2.0 mol/L in 0.25 mol/L intervals; Figure 7).

Both strains showed optimal growth and urease activity at 25 °C–30 °C (Figure 4). Notably, they remained metabolically active at 5 °C–15 °C, demonstrating psychrotolerance, although growth and urease activity were reduced. No activity was observed at 40 °C. The medium pH exceeded 9.0 across the 5 °C–35 °C range, indicating active urea hydrolysis (Figure 4e).

Optimal growth and urease activity were observed at an initial pH of 6–8 for both strains (Figure 5). They remained metabolically active at pH 4 and 10, albeit with reduced performance. The increase in medium pH from pH 4 to 10 confirmed continued urea hydrolysis (Figure 5e).

Both strains showed optimal growth and urease activity at 2% (*w*/*v*) NaCl (Figure 6). Strain L6 remained active at 6% NaCl, albeit with slower growth and reduced urease activity, whereas strain L8 exhibited no activity at this concentration.

Growth and urease activity were optimal in cultures with 0.25 mol/L urea (Figure 7). Both strains tolerated 0.75 mol/L urea, although growth and urease activity decreased at this higher concentration. Strain L6 showed higher urea tolerance compared with strain L8.

**Figure 8:**
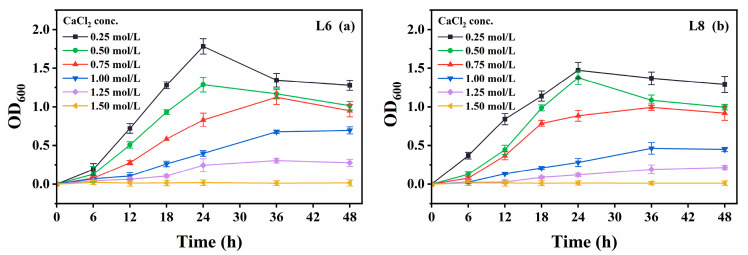
Effect of CaCl_2_ concentration on the growth of strains L6 and L8 (cultivation temperature, 30 °C; initial pH of the medium, 7.0; no urea was added to the cultures). (**a**) OD_600_ of strain L6; (**b**) OD_600_ of strain L8. OD_600_, optical density at 600 nm.

Growth was also measured in media containing various CaCl_2_ concentrations (0−2.0 mol/L; Figure 8). Urease activity was not measured due to CaCO_3_ precipitation upon urea addition, which could interfere with microbial propagation. Both strains showed optimal growth at 0.25 mol/L CaCl_2_ and tolerated up to 1.25 mol/L, albeit with slower growth.

The limestone mines of Mt. Buko, primarily comprising CaCO_3_ in the form of calcite, represent an alkaline, calcium-rich environment. Groundwater samples from the site show a pH of 8.0–8.5 and calcium concentrations of 0.82–1.45 mmol/L [40]. Given the mine’s location at an altitude of ~1000 m, the environment is also characterized by low salinity (less than 0.00003% NaCl) and low temperatures (annual average: ~10 °C). These results suggest that strains L6 and L8 are capable of sustaining metabolic activity and urease function under conditions reflective of their native habitat, supporting their potential application in MICP-based technologies for limestone mine environments.

### 3.4. Assessment of Ureolysis-Driven MICP by Strains L6 and L8 at Low Temperatures

**Figure 9:**
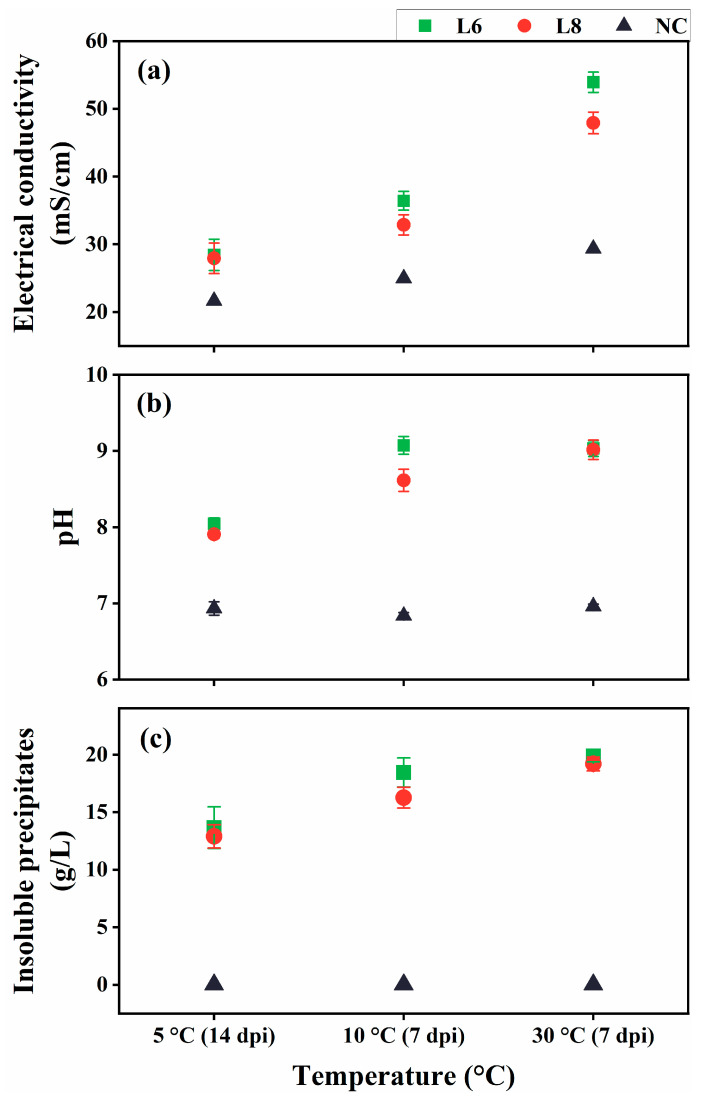
Ureolysis-driven MICP by strains L6 and L8 at 5 °C, 10 °C, and 30 °C. (**a**) EC, (**b**) media pH, and (**c**) precipitate formation. NC, negative control; dpi, days post-inoculation.

Given the psychrotolerant nature of strains L6 and L8, their ability to induce ureolysis-driven MICP was evaluated at low temperatures (10 °C and 5 °C; Figure 9). At 5 °C, minimal formation of insoluble precipitates was observed at 7 dpi, likely due to reduced metabolic activity. Therefore, the cultures at 5 °C were re-examined at 14 dpi. At all tested temperatures, the EC and pH of inoculated cultures were substantially elevated compared with uninoculated controls (Figure 9a,b), indicating active ureolysis, which led to increased ionic concentrations and media alkalinization. Notable insoluble precipitate formation occurred at 7 dpi for 10 °C and 30 °C cultures and at 14 dpi for 5 °C cultures (Figure 9c). No precipitates were detected in the uninoculated controls.

**Figure 10:**
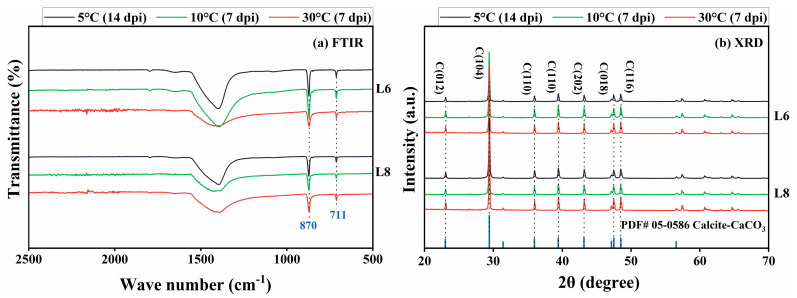
(**a**) FTIR spectroscopy and (**b**) XRD analysis of mineralization products at 5 °C, 10 °C, and 30 °C (a.u., arbitrary unit).

The precipitates were characterized using FTIR, XRD, and SEM/EDS. FTIR spectra revealed characteristic CaCO_3_ peaks at 870 and 711 cm^−1^ under 5 °C, 10 °C, and 30 °C conditions (Figure 10a). XRD analysis confirmed the formation of crystalline calcite at 5 °C, 10 °C, and 30 °C (Figure 10b), consistent with FTIR results.

**Figure 11:**
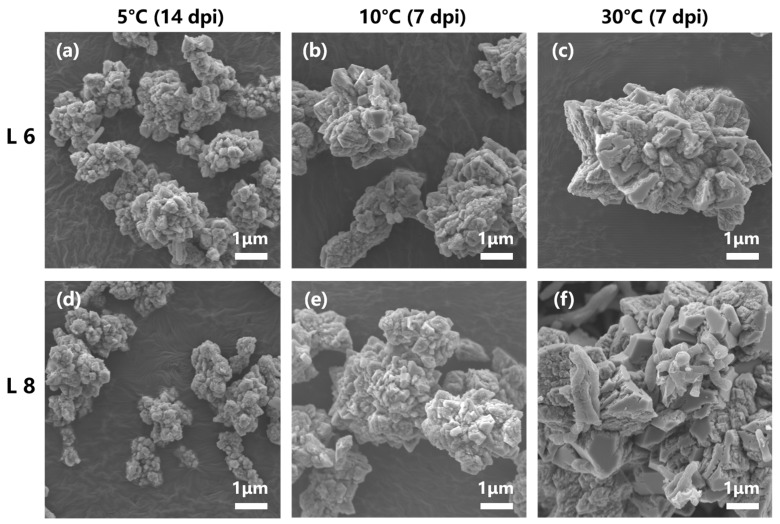
Scanning electron microscopy images of mineralization products formed at 5 °C, 10 °C, and 30 °C. (**a**–**c**) Mineralization by strain L6 at 5 °C (14 dpi), 10 °C (7 dpi), and 30 °C (7 dpi). (**d**–**f**) Mineralization by strain L8 at 5 °C (14 dpi), 10 °C (7 dpi), and 30 °C (7 dpi).

**Figure 12:**
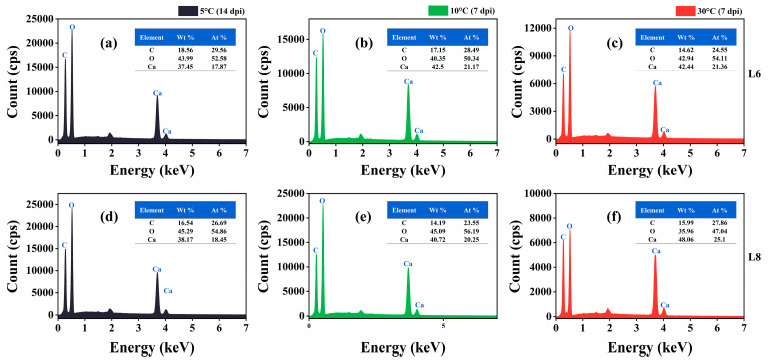
Energy-dispersive X-ray spectra of mineralization products at 5 °C, 10 °C, and 30 °C. (**a**–**c**) Mineralization by strain L6 at 5 °C (14 dpi), 10 °C (7 dpi), and 30 °C (7 dpi). (**d**–**f**) Mineralization by strain L8 at 5 °C (14 dpi), 10 °C (7 dpi), and 30 °C (7 dpi).

SEM images revealed the formation of scalenohedral crystals (typical calcite crystal form) at all tested temperatures (Figure 11). EDS showed that the constituent elements of the precipitates of strains L6 and L8 were C, O, and Ca, and the atomic content percentage (At.%) was about 1:3:1 (Figure 12), consistent with the elemental composition and proportion of CaCO_3_. These results indicated that CaCO_3_ crystals formed on bacterial surfaces, even at low temperatures. Thus, strains L6 and L8 induced ureolysis-driven MICP at low temperatures, matching the conditions at the high-altitude Mt. Buko limestone mine. However, a decrease in crystal size was observed with decreasing temperature, even with extended incubation at 5 °C (Figure 11), indicating that MICP occurs more slowly at lower temperatures.

**Figure 13:**
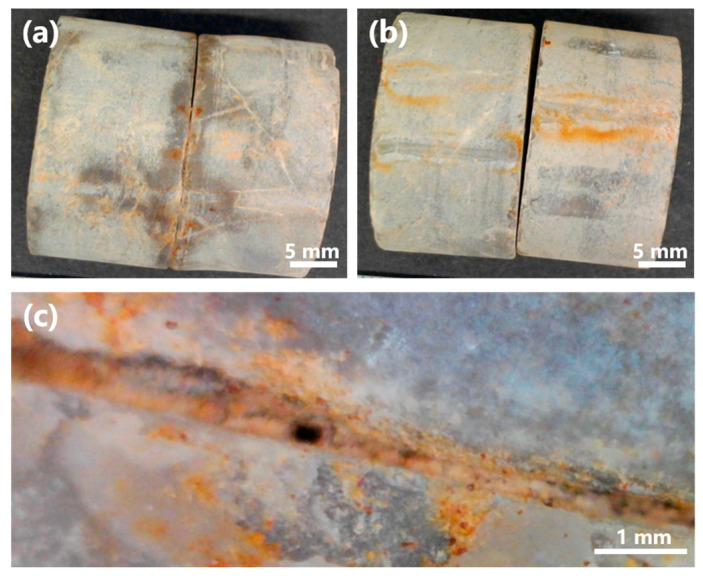
Rock fissure grouting test with strain L6. (**a**) Limestone cores grouted by strain L6 at 10 °C (14 dpi). (**b**) Control test without urea. (**c**) Magnified image of a sealed rock fissure.

To further evaluate the applicability of MICP in situ, a grouting test was conducted using strain L6 on limestone specimens obtained from Mt. Buko (Figure 13). Strain L6 was selected for the test, as it showed better characteristics than strain L8: strain L6 generally grew faster, reached higher OD_600_ (Figure 4, Figure 5, Figure 6, Figure 7 and Figure 8), and showed higher urease activity (Figure 4, Figure 5, Figure 6 and Figure 7) than strain L8 under most of the culture conditions; strain L6 showed MICP ability relatively better (higher EC, higher medium pH, and more precipitate production) than strain L8 at 10 °C (Figure 9). Pairs of limestone cores were assembled face-to-face to simulate rock fissures. The bacterium was inoculated into the simulated fissure and incubated for 14 d at 10 °C. In the presence of urea, strain L6 successfully grouted between the cores (Figure 13a,c), while no grouting was observed in the absence of urea (Figure 13b). These results suggest that strain L6 can seal cracks, fractures, or voids in limestone rock masses at low temperatures, likely by promoting CaCO_3_ precipitation on natural limestone surfaces via ureolysis. In a future study, the mineralogical composition of the precipitate and mechanism (including kinetics) of precipitate formation on limestone surfaces will be analyzed in more detail.

The splitting tensile strength of the grouted fissures was 8.1 ± 2.3 (standard error) kPa (*n* = 5). The relatively small adhesive strength may be attributed to the smooth, polished surfaces of the limestone specimens used in the test, as preliminary tests using unpolished fractured cores of limestone, sandstone, and andesite showed greater adhesive strengths (splitting tensile strengths of 29.4, 53.2, and 28.6 kPa, respectively), indicating that surface roughness likely plays an important role in MICP-based grouting efficiency.

## 4. Discussion

This study successfully isolated ureolytic bacteria from an active limestone mine. In a previous study, Omoregie et al. [41] isolated ureolytic bacteria from a limestone cave, namely, *Sporosarcina*, *Pseudogracilibacillus*, and *Bacillus* species capable of ureolysis-mediated MICP under ambient conditions (~30 °C), whereas our isolates were classified with *Rhodococcus* and *Bacillus*. Among them, *Rhodococcus* strains L6 and L8 exhibited tolerance to multiple environmental stressors relevant to conditions in their native limestone quarry and possible MICP applications. Notably, both strains were psychrotolerant and able to induce ureolysis-driven MICP at low temperatures reflective of the quarry’s high-altitude setting (~1000 m). To our knowledge, this is the first report of ureolysis-driven MICP occurring at 5 °C, representing the lowest temperature recorded for this process. Furthermore, strain L6 induced MICP at 10 °C on actual limestone surfaces, successfully sealing rock fissures. These findings suggest that indigenous microbial strains can remain metabolically active and facilitate MICP in situ within the limestone mine environment.

This study lays foundational groundwork for expanding MICP applications in limestone settings. Regarding such applications, Li et al. [26] demonstrated that *Sporosarcina pasteurii* could be used in conjunction with urea and glycerol as a dust suppressant in limestone mines. In this application, calcite deposition effectively bound dust particles, forming a stable calcite–dust cementation layer and enhancing dust suppression. MICP has also been proposed for stabilizing weathered limestone surfaces [42]. Surface weathering alters limestone mineralogy and structure, promoting rock embrittlement, erosion, rockfall, and slope failure. Instead of relying solely on conventional methods, such as anchoring or retaining walls, protective CaCO_3_ layers can be formed via photoautotrophic microorganisms. Field observations have confirmed the widespread occurrence of naturally formed carbonate coatings on weathered limestone outcrops, representing a natural analog of MICP-based limestone surface protection. Additionally, limestone waste from quarrying operations can be converted into Ca(CH_3_COO)_2_, which represents a calcium source (potentially superior to calcium chloride) for MICP applications [43].

MICP may serve as a supplementary technique for stabilizing rock slopes formed through decades of limestone mining. The Mt. Buko mine has been in operation for ca. 100 years, with more than 500 million tons of limestone extracted to date [44]. The bench cut method has resulted in a large, residual slope wall expected to reach 800 m in height and 5000 m in width by the end of operations. Given this scale, slope stability is a critical concern. The mechanical behavior of the rock slope is being monitored using several tools, including an automated polar system, a global positioning system, and crack gauges installed in an observation crosscut [45,46]. Extensive structural reinforcements, such as over 20 steel pipes (80 m long × 600 mm in diameter) and massive retaining rock piles (~2.4 million tons), are employed to maintain slope integrity. Groundwater and precipitation levels are also intensively monitored at the Mt. Buko site [39,46]. Water plays a critical role in determining the mechanical strength of rock masses. In limestone terrains, where rainwater infiltrates rapidly, slope stability is particularly sensitive to precipitation events. Previous studies have shown that groundwater levels are correlated with rock slope displacement: as levels decrease, slope movements are also reduced [40,47]. To mitigate water infiltration, haul roads on the slope surface are paved, and drainage holes have been drilled from the slope surface and observation drifts to reduce groundwater accumulation within the mine. As a supplementary strategy to these conventional measures, MICP holds promise for reducing the permeability of slope surfaces and reinforcing surface materials, thereby helping to prevent erosion and improve slope stability. Recently, field trials have demonstrated the applications of MICP in stabilization and erosion prevention of slope surfaces in various environments [48,49,50,51,52].

Future studies should evaluate ureolysis-driven MICP under conditions more representative of the in situ limestone mine environment, including a broader temperature range, varying oxygen gradients, media mimicking local groundwater chemistry, and implementation within native soils and rock matrices. Additionally, environmental microbiota are typically diverse and metabolically complex. Therefore, it is crucial to examine the potential ecological impacts of MICP applications. In particular, ureolysis generates ammonium as a byproduct, which can contribute to eutrophication, acidification of soil and water, and the release of toxic ammonia and nitrous acid. To minimize possible environmental harm, ureolysis-driven MICP may need to be applied in a spatially controlled manner. Alternatively, nonureolytic MICP pathways, e.g., photosynthesis-induced carbonate precipitation [42], could be employed.

## 5. Conclusions

This study evaluated the feasibility of MICP implementation in a limestone mine located at an elevation above 1000 m, which represents a low-temperature environment with an average annual temperature of ~10 °C. To this end, ureolytic bacteria were isolated from the actual limestone quarry. The representative isolates, *Rhodococcus* sp. strains L6 and L8, exhibited tolerance to key environmental factors relevant to MICP applications in the limestone-rich settings, including alkaline pH, high calcium levels, and elevated urea concentrations. Additionally, both strains were psychrotolerant, maintaining growth and urease activity at temperatures as low as 5 °C. Notably, both strains induced calcite crystal formation at 10 °C and 5 °C, although the reaction was slower at 5 °C. Furthermore, strain L6 demonstrated the ability to induce MICP on limestone surfaces, effectively sealing rock fissures. Thus, our results suggest that indigenous microbes retain metabolic activity in the limestone mine and are well suited for MICP applications.

## Data Availability

The partial 16S rRNA gene sequences of isolates L1–L8 have been deposited in the GenBank database under accession numbers PV855710–PV855717.

## Abbreviations

The following abbreviations are used in this manuscript:

EC	Electrical conductivity
EDS	Energy-dispersive X-ray spectroscopy
FTIR	Fourier transform infrared
MICP	Microbially induced carbonate precipitation
NC	Negative control
SEM	Scanning electron microscopy
XRD	X-ray diffraction
